# The role of trustworthy and reliable AI for multiple sclerosis

**DOI:** 10.3389/fdgth.2025.1507159

**Published:** 2025-03-24

**Authors:** Lorin Werthen-Brabants, Tom Dhaene, Dirk Deschrijver

**Affiliations:** SUMO Lab, IDLab, INTEC, Ghent University – imec, Ghent, Belgium

**Keywords:** artificial intelligence, multiple sclerosis, trustworthy AI, deep learning, uncertainty quantification

## Abstract

This paper investigates the importance of Trustworthy Machine Learning (ML) in the context of Multiple Sclerosis (MS) research and care. Due to the complex and individual nature of MS, the need for reliable and trustworthy ML models is essential. In this paper, key aspects of trustworthy ML, such as out-of-distribution generalization, explainability, uncertainty quantification and calibration are explored, highlighting their significance for healthcare applications. Challenges in integrating these ML tools into clinical workflows are addressed, discussing the difficulties in interpreting AI outputs, data diversity, and the need for comprehensive, quality data. It calls for collaborative efforts among researchers, clinicians, and policymakers to develop ML solutions that are technically sound, clinically relevant, and patient-centric.

## 1 Introduction

Machine Learning (ML) is increasingly applied to healthcare applications (1). While traditional statistical methods can help with biomarker discovery and recognizing trends and correlations, modern ML techniques such as Deep Learning (DL), are able to uncover complex correlations and provide better results than traditional, simpler techniques (2) due to their universal nature (3). Conversely, as these techniques become more complex, the need for *reliable* and *trustworthy* models increases (4, 5), especially within healthcare. However, building trust does not have a one-size-fits-all solution, resulting in many techniques to be developed to aid decision making.

For an end-user, be it a clinician or a patient, a model that is trustworthy is one that can provide certain guarantees on its predictions, explain its predictions, and provide a notion of uncertainty. For a complex disease such as Multiple Sclerosis (MS), the need for trustworthy models is especially pertinent, as its progression is non-trivially defined, and the decisions made to hinder its progression are important ones. A machine learning system that does not provide adequate reliability metrics, or trustworthy insights, will be less appealing to the end-user when there are high-stakes consequences. In recent years, the need for Trustworthy ML (TML) has also reached mainstream attention with the use of generative AI becoming more prevalent. For example, though Large Language Models have shown impressive results, they may still provide incorrect results, without any notion of uncertainty or trustworthiness (6). This is also known as the “hallucination” effect (7). Complex data and relationships warrant the use of trustworthiness techniques.

In the following Sections, we provide a summary of techniques present in Trustworthy ML (TML) (Section 2), why TML is necessary for MS (Section 3.1), and the associated challenges (Section 3.2).

## 2 Trustworthy machine learning

### 2.1 Out-of-distribution generalization

The many ways in which MS progression can occur (different limbs, locations of lesion growth, etc.), makes the disease variable and patient specific. Therefore, training data will rarely contain enough data to cover the full extent of the ways progression can be observed. Furthermore, due to protocols changing regularly and equipment variability, *concept* or *model drift* (8) may pose a real issue when ML models are deployed in the real world. Model drift occurs when new data do not correspond to the data on which a model was trained. As a result, models must be continually adapted so changes in data distributions are captured.

These issues can be tackled by making use of techniques such as domain adaptation (9, 10), a specific case of transfer learning (11), and synthetic data sampling such as SMOTE (12, 13).

The concept of Out-of-Distribution Generalization can be elucidated by considering a concrete example within the MS context. Imagine an ML model trained on data from North American patients. When this model is applied to patients from different geographical regions with distinct genetic and environmental factors, its predictions may falter due to differences in disease manifestation. Domain adaptation techniques can help here by adjusting the model to account for these regional variations. Similarly, synthetic data sampling, like the aforementioned SMOTE technique, can artificially—not necessarily in a representative way—augment the dataset to include underrepresented samples in a given dataset, improving the model’s robustness against a wide range of clinical scenarios. However, it must be stressed that data quality is key, and an underrepresented dataset can not fully capture the underlying factors to guarantee good out-of-distribution generalization.

### 2.2 Explainability and interpretability

A perfectly interpretable AI provides insights into the inner workings and decision process of an AI system. When it comes to the types of ML systems, they can broadly be divided into two categories: white-box models and black-box models.

#### 2.2.1 White-box models

Models that are inherently explainable and interpretable. These are often simpler methods such as linear or logistic regression, the latter of which can be represented as a nomogram (14), a graphical representation of such models that visually convey the weight of different input variables. These models can be fully dissected, so there may be many ways of representing or explaining them.

#### 2.2.2 Black-box models

Models that can not be interpreted easily, and are regarded as a “black box” out of which little or no knowledge can be derived. However, there are techniques that can provide explainability when working with black-box models, such as making use of Shapley values (15, 16) or making use of Deep Learning specific techniques (17) such as Layer-wise Relevance Propagation (18, 19). These are often post-hoc. In practice, these techniques will show a number of features and their importances expressed as a number. This could also be in the form of a heatmap. These feature importances may not always be as readily interpretable and may need training and education to comprehend adequately. Additionally, they do not necessarily *explain* why those features are important.

A classifier that may perform well in its evaluation metrics (sensitivity, specificity, ROC AUC, etc.) may still benefit from explainability methods. In particular, if models were to take into account many multimodal variables, the primary drivers of a given prediction may offer important insight for the user of the machine learning system.

Related to interpretable AI is explainable AI. Rather than being able to fully comprehend the inner workings of a model, an explainable AI model is able to be queried so that a reasonable explanation to the prediction is provided. Explainable AI can be viewed on different levels as well: Global, cohort, and local explainability. Global explainability provides information about the entire population or dataset. Due to the complex nature of the MS disease, valuable insights on a population level are scarce. Cohort explainability gives insight on subsets of the data, which can be more interesting when taking into account certain covariates. In this way, different groups of patients can be identified and correlations within these groups may offer more helpful insights than looking only at a global level. Lastly, local explainability provides insight on the model’s output for a single input example. Every patient has a different profile, and therefore local explainability may help acquire insight into the prediction of the model for that specific patient or observation.

### 2.3 Uncertainty quantification and calibration

#### 2.3.1 Uncertainty quantification

In machine learning models, uncertainty plays a critical, yet understated role in understanding and interpreting predictions. Healthcare specifically can greatly benefit from uncertainty quantification, as it can add a layer of trust between the user and the model (20–22). Two major sources of uncertainty are aleatoric and epistemic uncertainty (23).

##### 2.3.1.1 Aleatoric uncertainty

This type of irreducible uncertainty is inherent in the data itself. It cannot be reduced by adding more data and manifests as the noise within the data. An example of this uncertainty arises when using very few features. For example, a patient’s blood pressure is a crucial health metric, but it exhibits natural variability within an individual due to various factors like stress, activity level, time of day, and even the way it is measured.

This uncertainty can be either homoscedastic, when it remains constant for all values (e.g., base noise of a sensor), or heteroscedastic, when it varies depending on the value of the sample.

##### 2.3.1.2 Epistemic uncertainty.

Epistemic uncertainty arises from the model’s limited knowledge. This reducible uncertainty is high when the model has insufficient data to characterize or capture the target variable. Increasing the size of the data set can help reduce epistemic uncertainty. An intuitive example can be demonstrated as follows: Say there are multiple experts for a single disease such as MS. These experts may disagree on a given prognosis, despite all of them being equally trained for such a task. Analogously, in a machine learning model predicting patient outcomes for MS, the model might exhibit high epistemic uncertainty if it has been trained on a limited or non-representative dataset. Just as the disagreement among experts might stem from variations in their individual experiences and interpretations, the model’s uncertainty arises from its limited exposure to the diverse manifestations of the disease. By providing the model with more comprehensive data that captures a wider range of patient histories, symptoms, and outcomes, the epistemic uncertainty can be reduced, leading to more consistent and reliable predictions.

Applying uncertainty quantification in MS involves recognizing and managing the inherent unpredictability in patient responses and disease progression. For instance, a model expressing aleatoric uncertainty might show the variability in a patient’s symptoms over time, acknowledging that certain aspects of MS progression cannot be predicted with complete precision. Epistemic uncertainty can be illustrated by a model’s varying predictions based on different patient subgroups, reflecting limited knowledge about specific MS manifestations. To quantify and capture these uncertainties, techniques like Monte Carlo Dropout (MCD) (24) can be employed, providing a probabilistic understanding of a model’s predictions and helping clinicians make informed decisions under uncertainty.

Uncertainty quantification has been applied to lesion detection in MRI images (25–27), often making use of MCD or other methods of obtaining a model that can express uncertainty (28).

#### 2.3.2 Calibration

A well-calibrated machine learning model is one in which the model’s predicted probabilities closely match the probabilities observed in the actual data (29). Mathematically, this is represented as $P(y|\hat{\;p}(y)=\alpha )=\alpha$. This equation signifies that the probability of an event y occurring, given that the model predicts it with probability α, should ideally be α itself. As a practical example: a model that predicts the probability of 40% disease progression for a patient will ideally be correct 40% of the time of all patients who receive a similar prognosis. For methods such as neural networks, this is not often the case by default, and calibration needs to be improved. Additionally, calibration can also be applied to regressors that output a distribution, rather than a single value. In this case, the confidence interval (such as a 95% confidence interval, for example) can be calibrated to ensure that it matches the observations.

The need for calibration is evident in the lack of information an uncalibrated classifier or regressor provides. Often, as is the case with neural networks, a neural network classifier will collapse to output probabilities close to 100% or 0% consistently, rather than providing accurate probability estimates (29). As a result, a user of such a system needs to blindly trust the classifier rather than being able to take the confidence of the classifier into account.

## 3 Discussion

### 3.1 Why trustworthy ML is necessary for MS research

With the current knowledge of MS and performance of state-of-the-art machine learning models in the field, it stands to reason that there may not be a one-size-fits-all solution to detecting disease progression. Although other types of model (such as image classifiers) may perform very well and can reliably be used in most, if not all, cases, this may not be the case for MS. ML models for this purpose will likely be a tool to aid decision making, rather than a decision maker by itself. To that end, an ML model that just states “yes” or “no” is not sufficient. Rather, more information should be supplied to the user. A trustworthy version of this model will highlight parts of the input that contribute greatly to the prediction, show which global and cohort features are important, and also provide a notion of (un)certainty with the prediction. In this way, the user can:

• Select which predictions to trust and keep, both by using aleatoric and epistemic uncertainty as guides
• Analyze the subgroup in which the prediction fits
• Analyze the specific prediction and the features leading to the prediction

For MS research, the use and adoption of ML will be guided by advances in *trustworthy* ML. MS is a disease marked by its heterogeneity in symptoms, progression, and response to treatment, making reliable analysis of significant importance.

The ability of ML models to process and analyze different types of data—from clinical observations to MRI images—can lead to earlier detection and more precise monitoring of the disease’s progression. However, the value of these insights depends on their explainability. Clinicians and patients must be able to understand and trust the model’s predictions, necessitating a focus on explainable AI. For example, an ML model might identify subtle changes in brain lesions over time, but this information becomes clinically actionable only when it is presented in an understandable manner. Explainable models can elucidate the factors driving a prediction, thereby enhancing the clinician’s ability to make informed treatment decisions.

Moreover, the integration of uncertainty quantification in ML models is particularly relevant for MS. Given the variability in how the disease presents and progresses, models that can express their confidence in predictions are invaluable. They provide clinicians with a more nuanced understanding of each prediction, facilitating more informed risk-benefit analyses when deciding on treatment plans. A model that indicates a high level of uncertainty in its prediction might prompt further testing or closer monitoring, whereas a prediction made with high confidence could lead to more decisive action.

The importance of trustworthy ML in MS research also extends to patient empowerment. Access to understandable and reliable ML-driven insights can foster better patient-clinician dialogues. When patients understand the basis for predictions about their condition, they are better positioned to make informed decisions about their treatment and lifestyle choices.

### 3.2 Challenges of trustworthy ML for MS

#### 3.2.1 Integration of ML tools to aid clinical decisions

Integrating ML tools into existing clinical workflows presents another layer of complexity. For these tools to be adopted, they must fit into the highly regulated environment of healthcare. This integration involves designing user interfaces and metrics that are intuitive for clinicians, ensuring that ML predictions are presented in a way that complements decision-making processes rather than complicating them (30). Furthermore, imperfect data pose a problem during the training and prediction stages of an ML model. Data collection can be a laborious task, and in some cases the data cannot be accurately represented due to individual differences in disease expression. This rings especially true in the case of MS.

#### 3.2.2 Usability of uncertainty quantification and explainability techniques

As highlighted previously, UQ and explainability techniques have their merit, as they can highlight potential issues when making use of ML assisted decision systems. However, the end-user may not find much use in the way UQ results are represented in literature. Even explainabilty results have varying degrees of success concerning their usability (31). These techniques could benefit from user studies, as their usability hinges on the representation and, in turn, interpretation by the end-user. For example, rather than providing the clinician and/or patient with a numerical value signifying a “trustworthiness” score or certainty otherwise, larger trust could be gained by comparing the patient with other patients that have similar disease trajectories. This opacity can hinder trust and acceptance, especially in a high-stakes field like healthcare where understanding the “why” behind a diagnosis or prognosis is as crucial as the outcome itself (31).

#### 3.2.3 Out-of-distribution data, diverse data, available data

Data diversity and availability are critical factors that significantly influence the development and performance of ML models in MS research. MS is a disease with a highly variable clinical course and a wide range of symptoms that differ from patient to patient. This heterogeneity necessitates a rich and diverse dataset that captures the broad spectrum of the disease. After all, deep learning techniques are prone to overfitting, and may have performance below acceptable levels as a result (21, 32). Initiatives such as MSBase (33, 34) attempt to address the issue of out-of-distribution performance by providing multi-center data. The amount of data by itself may give the end-user a reason to trust a model, given enough diversity. Data quality is another concern, with issues such as missing values, inconsistent data entry, and the need for standardization across different data sources complicating the development of reliable ML models. Introducing diversity by including measurements that stray away from purely medical imaging or clinical data may also provide a new avenue of research, potentially discovering novel biomarkers. Future work should focus on developing models that can adapt to individual patient variations and incorporating emerging data types such as Motor Evoked Potentials (35, 36) into ML models.

## 4 Conclusion

This paper underscores the importance of trustworthiness in Machine Learning (ML) applications for Multiple Sclerosis (MS). Key aspects such as explainability, uncertainty quantification and calibration, and out-of-distribution generalization have been explored. Additionally, the challenges in integrating ML into clinical workflows and the hurdles posed by data diversity and availability have been discussed.

The authors urge the research community and healthcare providers to prioritize the development and implementation of trustworthy ML solutions for MS (and healthcare in general). There is an urgent need to foster partnerships between computer scientists, neurologists, and patients. This collaboration will ensure the development of ML solutions that are not only technically sound but also clinically relevant and patient-centric. Making comprehensive, high-quality data sets accessible while respecting privacy concerns is crucial. Initiatives should focus on standardizing data collection and sharing practices to aid in the development of more effective ML models. ML tools must be integrated into clinical workflows in a way that is intuitive and enhances decision-making processes. This involves designing user-friendly interfaces and ensuring that clinicians are adequately trained to use these tools effectively.

## Data Availability

The original contributions presented in the study are included in the article/Supplementary Material, further inquiries can be directed to the corresponding author.
